# Comprehensive Investigation of White Matter Tracts in Professional Chess Players and Relation to Expertise: Region of Interest and DMRI Connectometry

**DOI:** 10.3389/fnins.2018.00288

**Published:** 2018-05-03

**Authors:** Mahsa Mayeli, Farzaneh Rahmani, Mohammad Hadi Aarabi

**Affiliations:** ^1^Students' Scientific Research Center, Tehran University of Medical Sciences, Tehran, Iran; ^2^NeuroImaging Network (NIN), Universal Scientific Education and Research Network (USERN), Tehran, Iran

**Keywords:** chess, Raven's progressive matrices, diffusion MRI, connectometry, ROI, diffusivity

## Abstract

**Purpose:** Expertise is the product of training. Few studies have used functional connectivity or conventional diffusometric methods to identify neural underpinnings of chess expertise. Diffusometric variables of white matter might reflect these adaptive changes, along with changes in structural connectivity, which is a sensitive measure of microstructural changes.

**Method:** Diffusometric variables of 29 professional chess players and 29 age-sex matched controls were extracted for white matter regions based on John Hopkin's Mori white matter atlas and partially correlated against professional training time and level of chess proficiency. Diffusion MRI connectometry was implemented to identify changes in structural connectivity in professional players compared to novices.

**Result:** Compared to novices, higher planar anisotropy (CP) was observed in inferior longitudinal fasciculus (ILF), superior longitudinal fasciculus (SLF) and cingulate gyrus, in professional chess players, which correlated with higher RPM score in this group. Higher fractional anisotropy (FA) was observed in ILF, uncinate fasciculus (UF) and hippocampus and correlated with better scores in Raven's progressive matrices (RPM) score and longer duration of chess training in professional players. Consistently, radial diffusivity in bilateral IFOF, bilateral ILF and bilateral SLF was inversely correlated with level of training in professional players. DMRI connectometry analysis identified increased connectivity in bilateral UF, bilateral IFOF, bilateral cingulum, and corpus callosum in chess player's compared to controls.

**Conclusion:** Structural connectivity of major associational subcortical white matter fibers are increased in professional chess players. FA and CP of ILF, SLF and UF directly correlates with duration of professional training and RPM score, in professional chess players.

## Introduction

White matter fibers undergo extensive changes during critical periods of maturation (Cao et al., 2016), during long-term practice in complex interpretive tasks (Zuraw et al., 2013), and later during aging. Understanding the neural basis by which human brain can make adaptations during mastership challenges has long been an issue of interest.

Chess playing has been associated with several cognitive benefits, including improvement in learning transfer to mathematical literacy and problem-solving in children at school age (Sala and Gobet, 2016). Chess is also known to improve cognitive reserve in old age (Yang et al., 2016). It is known that chess playing requires expertise in various cognitive tasks, including object perception, pattern recognition, fluid reasoning and speed processing (Burgoyne et al., 2016). Understanding the basis of these alterations has always been a subject of research.

Conflicting results suggest that expertise in cognitive tasks could either result from inherent structural differences between experts and non-experts or result from axonal rearrangements during adaptive changes that happen with long-term training (Oechslin et al., 2009). Early studies focused on morphometric evaluations of brain regions and yielded interesting results. Increased gray matter volume was observed in motor planning areas of frontal cortex in Judo players, and in hand motor and dexterity region of precentral gyrus in musicians (Wan and Schlaug, 2010). Interestingly, these structural changes correlated with age of initiation of training and with training time spent on music or sport (Chang, 2014) and also with the age of onset of learning in linguistic skills (Rahmani et al., 2017).

Studies have addressed functional changes in brain's regions involved in various cognitive processes, as a result of long-term training in chess (Lee et al., 2010; Duan et al., 2012; Fattahi et al., 2015). Enhanced functional interconnectivity of neural networks involved in attention, visuospatial processing, and problem-solving have been revealed in professional chess players compared to novices (Duan et al., 2014). This happens with a concomitant strong suppression of default mode network and engagement of task-specific networks in “master” and “grand master” players (Atherton et al., 2003).

One of the few studies using diffusion tensor imaging (DTI) in musicians, revealed increased fractional anisotropy (FA) in cross-hemispheric white matter connective fibers such as corpus callosum in musicians (Schmithorst and Wilke, 2002). Musicians who started at early age were more likely to display these structural reorganizations (Chang, 2014), confirming that the observed changes were experience-dependent (Chang, 2014). Interestingly, functional connectivity was found to be increased in sensorimotor, executive and auditory networks (Luo et al., 2012), while DTI revealed the changes to be most prominent in corpus callosum and next in non-motor dependent areas (Schmithorst and Wilke, 2002). This represents a higher sensitivity for diffusion MRI in identification of adaptive microstructural changes associated with expertise and suggests that diffusion MRI might better reveal white matter areas in which adaptive changes correlate better with time spent on training and level of expertise.

Based on the functional networks identified in chess players, Hanggi and his colleagues attempted to determine differences in volume and cortical thickness of frontal and parietal areas in chess players, followed by analysis of diffusion tensor metrics of white matter tracts (Hanggi et al., 2014). This limited DTI result disclosed increased mean diffusivity in left superior longitudinal fasciculus (SLF), a subcortical white matter fiber with implications in spatial recognition, in chess players (Hanggi et al., 2014). Compared to conventional extraction of DTI metrics using end-to-end fiber tracking, diffusion MRI (DMRI) connectometry provided higher sensitivity and increased analysis power in identifying microstructural differences in white matter between two study groups. Using the notion of “local connectomes” (Yeh et al., 2016), DMRI connectometry adopts a new approach in tracking differences of structural connectivity patterns between study groups or investigating correlation of these microstructural changes considering any variable of interest (Yeh, 2017). DMRI connectometry might provide a better tool to identify neural underpinnings of chess-expertise, based on differences in structural connectivity in professional players and novices. Whether these areas conform to those in which diffusion metrics correlate with amount of chess expertise and playing, can confirm the above mentioned hypothesis.

Using the dataset deposited by Li et al. (2015), at: doi: 10.15387/fcp_indi.pro.wchsuchess, we adopted a step-wise analytic approach to identify: (i) between group differences in diffusivity variables, extracted from areas defined by Johns Hopkins University's Mori white matter atlas, (ii) correlation of these diffusometric values with measurements of chess expertise/training in professional players using partial correlation analysis, proceeding to confirm the results of these two approaches with (iii) DMRI connectometry to identify areas with difference in connectivity between professional players and novices.

## Experimental procedure

### Participant and phenotypic data

The study sample consisted of 29 professional chess players (age: 28.7 ± 10.8; 9 females) and 29 age matched novices (age: 25.7 ± 6.9; 15 women), with Chinese origin. Chess players and novices were matched in their age, sex and education years (*p* = 0.11, 0.22, 0.41 respectively). Professional chess players were all under intensive training (mean training time: 4.24 ± 1.73 h/day) and scored on average 2401.09 ± 134.58 on Elo's professional chess-skill rating. Six participants from the professional chess players were rated as grand masters (GM) and 11 as masters (M) and the rest of chess players (#12) were either in level 1 or 2 of chess playing (lower levels). Twenty-three of the 29 professional players scored above the standard United States Chess Federation's entry level for chess masters. Regarding chess skills in the novice players, 11 participants from this group declared that they used to play chess and another 11 only knew the rules of chess playing, five participants from the novices did not know the rules of chess playing nor had ever played chess and only two novices played chess often. Participants in both groups were all right-handed, with no recorded history of relevant clinical disorders.

The original database from Li et al. also entails years of official education, nationally-certified game rating scores in 2009, as well as age of start of chess practice, duration of chess practice per day, week, month and year in professional players. The Raven's Progressive matrices (RPM) test (Raven, 1998) score is also included in the database for all participants. Professional chess players scored on average 49.03 ± 6.6 on RPM test, had started playing on 8.5 ± 2.8 years and had started professional training at a mean age of 17 ± 5.8 years old (Li et al., 2015).

The Research Ethics Committee of West China Hospital of Sichuan University approved the protocol of acquisition of the multimodal MRI dataset of professional chess players used by this article. Also, informed consents were collected from participants prior to data acquisition (Li et al., 2015).

### Data acquisition

Three sessions of MRI data were collected from a Siemens 3T TRIO system (Siemens, Erlangen, Germany) at West China Hospital of Sichuan University, Chengdu, China for each subject. First, a 1 mm isotropic high-resolution T1-weighted structural image or sMRI was acquired using an MPRAGE sequence. Imaging parameters were as follows: *TR* = 1,900 ms, *TE* = 2.26 ms, *TI* = 900 ms, Bandwidth = 200 Hz/Px, Field of view = 256^*^256 mm2, Flip angle = 9°, and 176 slices.

Utilizing single shot echo planar imaging (EPI) sequence, two runs of diffusion MRI data were collected. For each run, one b0 image and 20 diffusion-weighted images were acquired. The parameters were: *TR* = 6,800 ms, *TE* = 93 ms, Bandwidth = 1,396 Hz/Px, Fov = 230^*^230 mm2, and Voxel size = 1.8^*^1.8^*^3.0 mm3 (Li et al., 2015).

### Diffusion MRI data preprocessing and ROI analysis

Diffusion MRI data were corrected for subject motion, eddy current distortions and susceptibility artifacts due to the magnetic field inhomogeneity, using Explore DTI toolbox (Leemans et al., 2009). Image corrections consisted of the following steps: (i) correction for subject motion and eddy current induced distortions (Klein et al., 2010); (ii) tensor estimation using the iteratively reweighted linear least squares estimation (Veraart et al., 2013); and (iii) automated atlas-based analysis with the Johns Hopkins University's Mori white matter atlas using affine and elastic registration based on “elastics.” After these preprocessing steps, fractional anisotropy (FA), axial diffusivity (AD), radial diffusivity (RD), mean diffusivity (MD), and planar anisotropy [Cp (planar tensor)] values were calculated for each of the 20 white matter brain regions provided by the Mori atlas, and for each subject.

Three cases were excluded from analyses due to low-quality measures based on: (i) average residuals per DWI or (ii) across DWI for each voxel and/or (iii) absence of corpus callosum (CC) in visual check. Visual quality control procedures were also performed, i.e., image reading of the T1WI to rule out significant brain abnormalities, including excessive white matter lesions, silent brain infarction, and hydrocephalus. None of the subject in this study had white matter lesions to the extent that they required exclusion.

### Connectometry analysis

Diffusion MRI connectometry was used to study the differences in microstructural connectivity between two groups (Yeh et al., 2016). To attain the spin distribution function (SDF), diffusion data were reconstructed in the MNI space using q-space diffeomorphic reconstruction (QSDR) (Yeh and Tseng, 2011). The diffusion sampling length ratio of 1.25 was used, and the output resolution was set on 2 mm. QSDR is a model free resolution to obtain the SDF value from DMRI signals, in a standard space (Yeh, 2017). As QSDR uses a flexible reconstruction procedure it can be implemented on various diffusion sampling outlines and also in various standard spaces. The SDF is a numeric measure of the density of water diffusion and is calculated for any given direction of a voxel following QSDR reconstruction. QSDR preserves five directions and orientations from the MNI atlas along with spin distribution, which are then used to make statistical deductions. SDF is later converted to quantitative anisotropy (QA), which is a new diffusometric parameter correlating with the peak value of SDF for each fiber orientation (Yeh, 2017).

The SDF values were used in the connectometry analyses in the next step. A threshold of 2 was assigned for selecting local connectomes, and significant tracts were identified using a deterministic fiber tracking algorithm (Yeh et al., 2013). A 40 mm length threshold was used to select tracts. The seeding density was 50 seeds per mm^3^. To estimate the false discovery rate, a total of 2000 randomized permutations were applied to the group label to obtain the null distribution of the tract length. The analysis was conducted using DSI Studio (http://dsi-studio.labsolver.org).

### Statistical analyses

We analyzed the group data using SPSS for Windows version18.0 (SPSS, Inc., Chicago, IL). We checked the normality of our data using the Shapiro-Wilk test, for age, sex distribution, RPM score, age of onset of training, professional training time in years, and level of training.

For the first part of the analyses, two-tailed independent sample *t*-tests, and if indicated Fisher's exact test were used to examine group-differences between professional chess players and novices in: (1) age, sex and education years and (2) diffusometric variables FA, AD, RD, and MD from tracts defined by Mori white matter atlas. These included; bilateral anterior thalamic radiations, bilateral corticospinal tracts (CST), bilateral cingulate gyri (CG), bilateral hippocampus, forceps major, forceps minor, bilateral inferior fronto-occipital fasciculus (IFOF), bilateral inferior longitudinal fasciculus (ILF), bilateral superior longitudinal fasciculus (SLF), bilateral uncinate fasciculus (UF) and bilateral temporal parts of superior longitudinal fasciculus (temporal-SLF). Significant results were corrected for false positive (type I error) tracts using Bonferroni correction of raw *p*-values (Table 1).

**Table 1:** Differences in diffusometric values of gray and white matter structures based on Mori atlas.

		Professional players^*^	Novices^*^	*P*-value^**^
Cingulate gyrus (Right)	Planar anisotropy	0.139 ± 0.008	0.135 ± 0.009	0.049
Cingulate gyrus (Left)	Planar anisotropy	0.145 ± 0.006	0.140 ± 0.01	0.017
Hippocampus (Left)	Planar anisotropy	0.114 ± 0.008	0.108 ± 0.01	0.046
	Fractional anisotropy	0.184 ± 0.013	0.173 ± 0.019	0.019
Inferior fronto-occipital fasciculus (Right)	Planar anisotropy	0.136 ± 0.005	0.130 ± 0.008	0.008
Inferior fronto-occipital fasciculus (Left)	Planar anisotropy	0.136 ± 0.005	0.131 ± 0.008	0.021
Inferior longitudinal fasciculus (Right)	Planar anisotropy	0.127 ± 0.006	0.123 ± 0.009	0.046
	Fractional anisotropy	0.208 ± 0.01	0.199 ± 0.017	0.03
Inferior longitudinal fasciculus (Left)	Planar anisotropy	0.132 ± 0.005	0.127 ± 0.01	0.037
	Fractional anisotropy	0.206 ± 0.008	0.197 ± 0.016	0.02
Uncinate fasciculus (Right)	Planar anisotropy	0.120 ± 0.007	0.114 ± 0.008	0.002
	Fractional anisotropy	0.211 ± 0.008	0.204 ± 0.014	0.027
Superior longitudinal fasciculus left temporal part	Planar anisotropy	0.150 ± 0.011	0.142 ± 0.010	0.005

* No significant difference was observed in sex ratio, age and years of education between professional players and novices

** *only significant results are shown for between group differences in diffusion values of fibers*.

**Figure 1:**
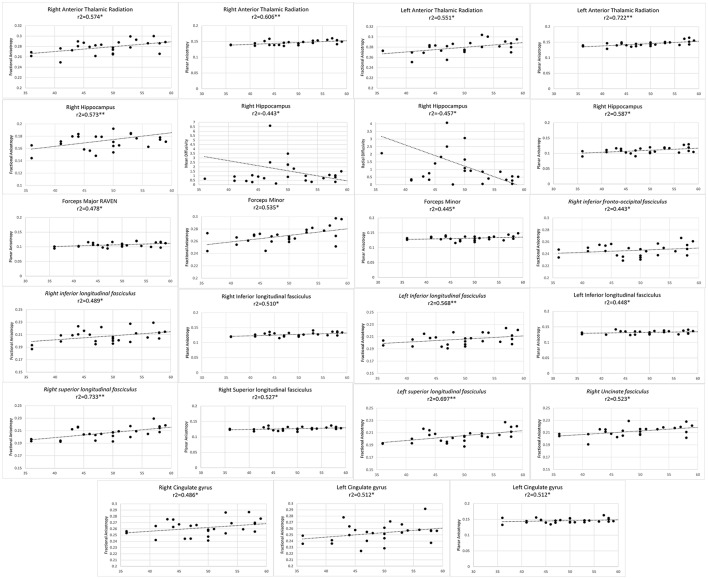
Significant results from partial correlation analysis of diffusion metrics of Mori white matter atlas and chess players score in Raven's progressive matrices test.

**Figure 2:**
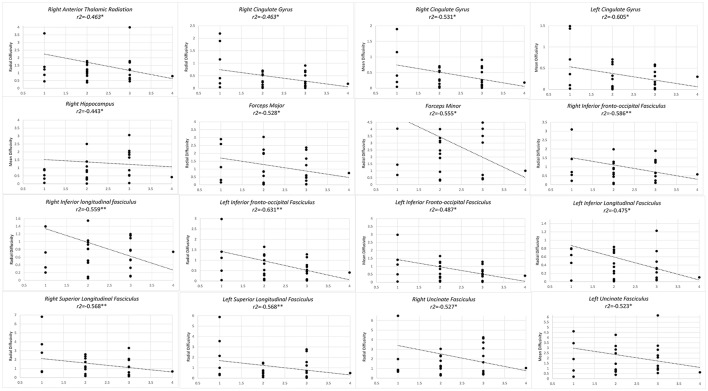
Significant results from partial correlation analysis of diffusion metrics of Mori white matter atlas and chess player's level of mastership.

**Figure 3:**
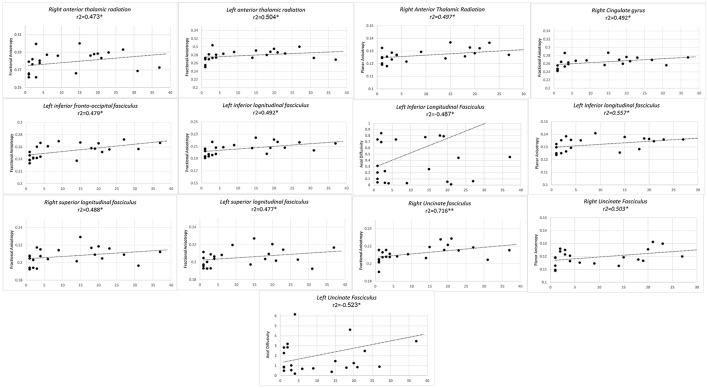
Significant results from partial correlation analysis of diffusion metrics of Mori white matter atlas and chess player's professional training time in years.

Finally, average value of FA, AD, RD, and MD metrics were partially correlated against professional training time, level of play (level 1, 2, master or grand master) and total score in RPM. Participant's years of education, age and sex were considered as covariates. Permutation testing was conducted to compensate for type I error inflation as a result of multiple comparisons in the correlation matrix (Figures 1–**3**).

## Results

### Between-group analyses

As a first step, we investigated whether diffusometric variables of the areas defined by Johns Hopkins University's Mori white matter atlas showed a difference between professional chess players and novices. No significant difference was observed in age, sex and years of formal education between professional players and beginners (Table 1; *p* = 0.11, 0.22, 0.41 respectively). Higher mean planar anisotropy (CP) was seen in bilateral CG, bilateral ILF, and bilateral IFOF and also in left hippocampus, temporal part of the left SLF, and right UF, in professional players compared to novices (Table 1). A concomitant increase in FA was observed in the left hippocampus, bilateral ILF, and left UF (Table 1), consistent with adaptive maturation in white matter tracts.

### Partial correlation

Next step, through Pearson's partial correlation, we investigated whether AD, MD, FA and RD values of the 20 regions defined by the Mori atlas could be predicted by variables related to chess proficiency: total training time in years, score in RPM test, or level of the mastership (level 1, level 2, master and grandmaster) in professional players (Figures 1–**3**). We controlled the results for confounding effect of age, sex and years of formal education of chess players. Type I error inflation was controlled using permutation testing.

As professional chess players scored higher in RPM test, higher FA values were observed in bilateral anterior thalamic radiations, bilateral CG, bilateral ILF, bilateral SLF, forceps minor, right UF, right hippocampus, and right IFOF. Positive correlation observed with FA in the right hippocampus and RPM score, was concomitant with significantly lower MD and RD values. Planar anisotropy of bilateral anterior thalamic radiations, bilateral ILF, forceps minor and major, right SLF and right hippocampus as well as left CG directly correlated with RPM score (Figure 1).

Higher level of training from level 1 to level 2, from master to grand master, correlated with lower RD in forceps major and minor, bilateral IFOF, bilateral ILF, bilateral SLF as well as right UF and right CG. Similarly a negative correlation was observed in MD of bilateral CG, left UF and left IFOF (Figure 2).

Finally, longer duration of professional training predicted higher FA values of bilateral anterior thalamic radiations, bilateral SLF, left ILF, left IFOF, and right UF and right CG. In left ILF a concomitant inverse correlation was observed with AD along with a positive correlation with CP. Higher CP values also concurred with higher FA, with an increase in duration of professional training in right anterior thalamic radiations and right UF (Figure 3).

### Diffusion MRI connectometry

**Figure 4:**
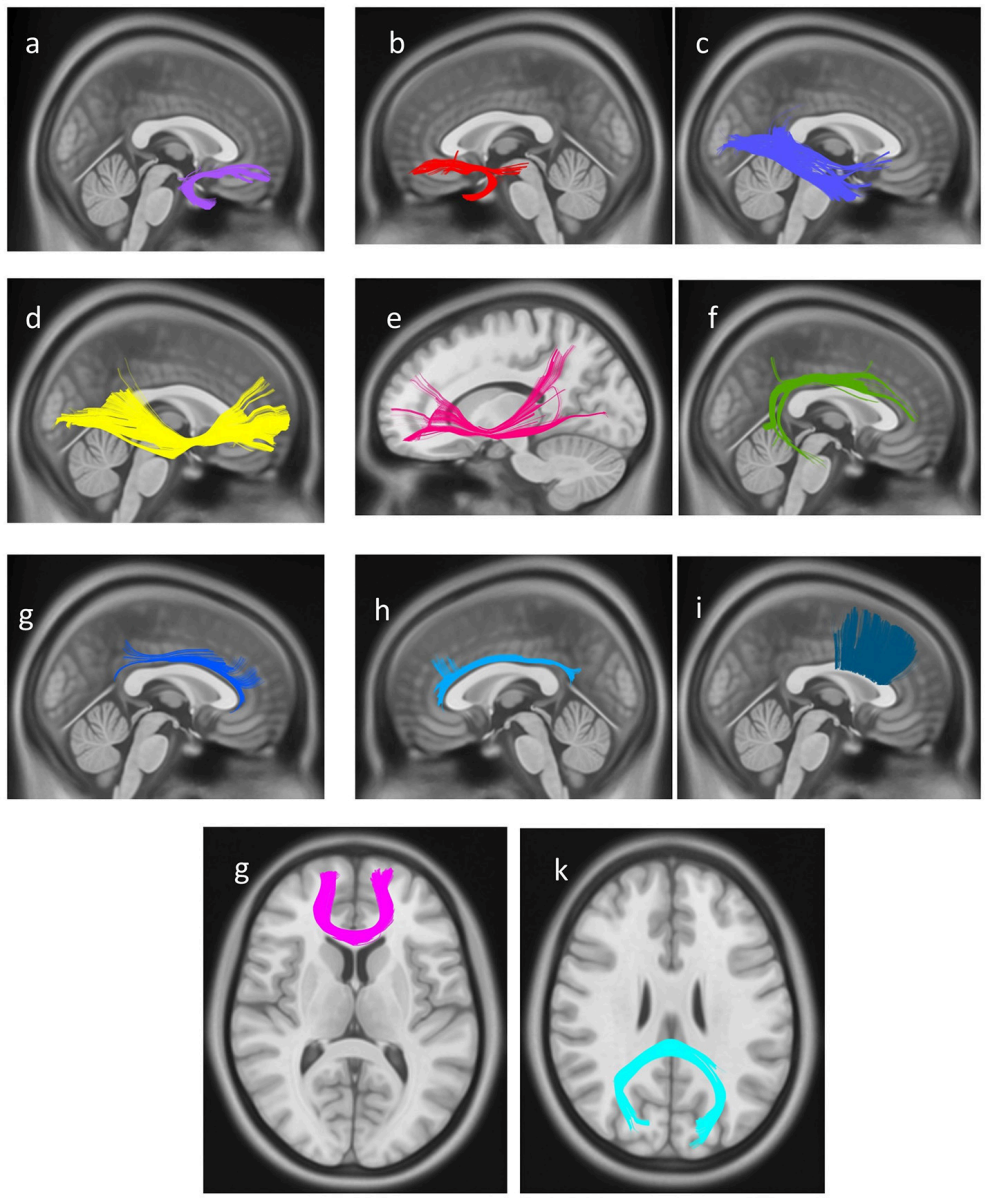
White matter pathways with significantly increased connectivity in chess player's compared to healthy controls (*FDR* = *0.0285*), **(a)** right uncinate fasciculus, **(b)** left uncinate fasciculus, **(c)** right inferior longitudinal fasciculus, **(d)** right Inferior fronto-occipital fasciculus, **(e)** left Inferior fronto-occipital fasciculus, **(f)** right arcuate fasciculus, **(g)** right cingulum, **(h)** left cingulum, and **(i)** body of corpus callosum, **(g)** genu of corpus callosum, **(k)** splenium of corpus callosum.

In the final step, a connectometry approach was implemented to identify differences in white matter connectivity between the professional chess player and novices in a hypothesis-free manner. The density of diffusing spins at various orientations was calculated, and diffusion MRI connectometry was conducted using a *t*-test. The analysis identified increased connectivity (*FDR* = *0.0286*) in bilateral UF, bilateral IFOF, bilateral cingulum, right arcuate fasciculus (AF), and genu, body, and splenium of CC, in professional players compared to controls (Figure 4).

## Discussion

We extracted diffusion metrics from 20 white matter regions, through ROI analysis of John Hopkins Mori atlas. First, we sought to identify possible microstructural differences in white matter based on diffusivity parameters, FA, AD, MD, RD, and CP, between professional chess players and novices. Next, we partially correlated the diffusion metrics for each area with professional training time, level of mastership, and RPM test scores, in professional players. The goal of this step was to investigate whether these structural adaptations correlated with training time or level of mastership in professional players. Finally, and to confirm results of between-group and correlation analyses, we took advantage of a hypothesis-free approach in DMRI connectometry, to see whether the same white matter areas showed differences in structural connectivity in professional players compared to novices.

Results of the first step confirmed structural adaptations in white matter in professional players but did not disclose whether these changes related to chess training or level of mastership or possibly correlated with general reasoning capabilities of the chess players, as measured by RPM test. Many of the white or gray matter regions that showed increased fiber integrity, in terms of CP and FA, showed positive correlation with years of chess training or RPM test. CP of left cingulate gyrus, bilateral ILF and right UF was higher in professional players and directly associated with RPM score and training time respectively. Similarly, FA of bilateral ILF was higher in professional players and directly correlated with RPM score. This happened with a concomitant inverse correlation of radial diffusivity of these fibers and level of training in professional players.

As it appears, not all the diffusometric parameters of the investigated regions in first step, show significant results in correlation analysis. The reason for this is 2-fold: first, different sensitivity of diffusion parameters in detecting microstructural changes in different regions. This is primarily based on the sensitivity of each of the diffusion metrics to detect physiological vs. pathological changes in white matter integrity and secondly relies on the difference of anatomical orientation in fibers/regions. Radial and axial diffusivity are sensitive markers of fiber demyelination or axonal damage. While the relative changes in RD and AD, during maturation and adaptive changes in white or gray matter is still unknown. On the other hand, higher FA and lower MD values indicate fiber integrity and health. Structurally intact white matter fibers are expected to have higher FA values and lower MD. Changes in these two parameters also happen during white matter maturation during adolescent (Lebel et al., 2008). It should be noted that planar anisotropy is a more valid parameter in areas with expanding or fanning out of fibers, while axial diffusivity in more sensitive to measure integrity along the main axis of a fiber. Finally, FA can give information about the overall organization of the fiber in all directions of tensors.

Second reason for the discordance between fibers identified in each steps relies in the sensitivity of analyses in each step. Diffusivity in some fibers show significant correlation with chess training and expertise but the sum of difference in fiber's micro organization, induced by chess training, is not enough to yield significant results when compared to novices. In other words, some fibers undergo relevant changes in chess players but not enough to make statistical difference in diffusivity with novices, yet enough to show microstructural differences.

In our first analysis, many subcortical associational white matter tracts showed higher planar anisotropy, while some depicted concomitant increase in FA (Table 1). CP value gives a measure of water molecules tendency to diffuse over two-dimensional sections of the fiber (Yeh, 2017). Measurement of planar anisotropy in white matter fibers with a high number of crossing fibers gives a more precise measure of fiber integrity than other diffusometric variables. High CP can also reflect an increase in FA when fibers tend to fan out from their anteroposterior direction (Tournier et al., 2011), as happens in our results with temporal part of the left SLF. This could also explain why increased CP values concurred with higher FA values in left hippocampus, left uncinate fasciculus and bilateral ILF (Table 1). High CP can also be seen when white matter areas undergo both expansion and maturation, as happens during adolescence (Qiu et al., 2008). Higher planar anisotropy and fractional anisotropy of areas of chess players is in line with the results of the next step, in which diffusometric values of the same areas positively correlated with more years spent of training and higher level of expertise in chess. This could also suggest a benefit for maturation of cognitive abilities in children who have undergone early chess training, concomitant with the observed changes in white matter fiber integrity (Lebel et al., 2008).

One study found similar results in correlation of diffusometric parameters of chess players confined to right SLF. Higher chess players' Elo score and lifetime hours of chess training robustly correlated with lower MD and AD values in SLF (Hanggi et al., 2014). Another study reported in a study on professional concert pianists, showed a direct correlation between the sum of hours practiced on piano playing and FA in the corpus callosum and arcuate fasciculus (SLF part IV) (Bengtsson et al., 2005). Similarly, we found that the FA of the SLF could be predicted by training time and chess player's score in RPM test.

When correlating diffusometric values with level of chess training, direct correlation of regions CP was observed, concomitant with inverse correlation of MD or RD value in several fibers including; bilateral anterior thalamic radiations, forceps minor and major, bilateral ILF, right SLF, right hippocampus, and left cingulate gyrus. This confirms the overall microstructural reorganization of these fibers in favor of increased structural integrity.

Diffusivity in bilateral IFOF, bilateral cingulate gyri, bilateral UF and bilateral anterior thalamic radiations have been shown to predict higher fluid reasoning ability, a key competency in chess, in healthy adolescents (Ferrer et al., 2013). This is in line with our finding on positive correlation of RPM scores with FA and CP in these fibers (Figure 1). RPM is a classic test, designed in 1938 as “a test of a person's present capacity to form comparisons, reason by analogy and develop a logical method of thinking regardless of previously acquired information” (Raven, 1938; Sternberg, 1977). Chess players have higher processing speed and reasoning abilities (Grabner et al., 2006), and our results put further spin that the evolution of this ability correlates with adaptive changes in the microstructure of the mentioned fibers.

Finally, DMRI connectometry confirmed increased connectivity in bilateral IFOF and bilateral UF, compatible with the results of between-group and correlation analyses (Figure 4). Connectometry uses SDF as the primary function. SDF calculates the degree of water density in all diffusion spins of water molecules, in any given voxel direction (Yeh et al., 2016). By measuring the peak amount of SDF for each given fiber orientation, connectometry identifies areas with similar patterns of connectivity or “local connectomes.” This produces another metric called QA. Diffusion MRI connectometry reconstructs local patterns of connectivity within adjacent voxels of a white matter fascicle, and maps concordance of this local connectivity patterns with a variable of interest, along the length of a fiber. By measuring distribution density rather than diffusivity, connectometry circumvents the problem of statistical deduction based on average diffusivity value, in areas with kissing or crossing fibers. In these areas, conventional analyses like ROI based statistics fail to produce results with high statistical power. Connectometry based on local connectome patterns is also more spatially specific than voxel-based analyses (Yeh et al., 2016). Finally, QA is overall more sensitive to white matter local pathology while FA and conventional diffusion metrics better predict along-tract pathologies of white matter fibers (Yeh et al., 2016).

In the following sections we procced to describe relevance of UF, IFOF, and ILF that showed difference between groups in both between-group ROI analyses and connectometry approach. We also describe relevance of SLF which had shown significance with Elo score in previous studies (Hanggi et al., 2014), as well as corpus callosum as the largest commissural fiber in the brain. CC showed increased connectivity in chess players in our study, and had previously been correlated with training hours in pianists (Bengtsson et al., 2005).

### Uncinate fasciculus

UF serves as a bidirectional connection between lateral orbitofrontal cortex, the anterior-most part of the prefrontal cortex with the anterior temporal lobe (Von Der Heide et al., 2013). Heide and his colleagues proposed a role for uncinate fasciculus in conditional rule learning i.e., learning responses associated with reward based on cues or patterns recognized by temporal lobe (Von Der Heide et al., 2013). Interestingly, UF is among fibers with the unusual developmental peak at the third decade of life (Voineskos et al., 2012). It is therefore plausible to deduce that the microstructure of UF is affected by lifelong chess exercise or other activities that entail pattern recognition tasks.

Anisotropy of the uncinate fasciculus is higher in right side (Luders et al., 2011), and UF was thus initially considered as a language related area. Later a role in auditory-verbal memory and in pattern-based decision-making situations is proposed for UF on both sides (Von Der Heide et al., 2013), contrary to the first hypothesis. In a cross-sectional analysis increased structural connectivity and higher FA value in left temporal SLF, and bilateral UF was detected in meditation practitioners (Luders et al., 2011). Abnormalities in left UF are associated with socioemotional deprivation in children (Eluvathingal et al., 2006) and left UF is structurally damaged in behavioral variant of frontotemporal dementia (Rohrer, 2012). Our results revealed that diffusivity variables in right uncinate fasciculus showed correlation with level of training and with time spent on training in professional players, possibly conferring task-related pattern recognition benefits in chess players. We also demonstrated increase connectivity in bilateral UF in professionals, which could be interpreted in the light of socioemotional enrichment of young chess players, compared to their peers (Aciego et al., 2012).

### Inferior fronto-occipital fasciculus

IFOF is the longest associative bundle in the human brain, extending from occipital lobe, to temporo-basal areas and to superior parietal lobule and frontal lobe (Almairac et al., 2015), justifying its integrative role and general semantic function (Martino et al., 2010). Also, a decline in IFOF integrity happens with age-related decline in face recognition, consistent with the integrative role of IFOF in visual memory and visuospatial skills (Thomas et al., 2008). Results of a meta-analysis published in 2016, revealed that the relation between chess skills and cognitive ability is most robust in numerical abilities, followed by verbal abilities, while visuospatial skills showed only week correlation (*r* = 0.13), with chess skills (Burgoyne et al., 2016). Interestingly, association of visual integrative abilities with chess expertise was even weaker in adult professionals. Therefore, if IFOF is implicated in chess-plyers brain adaptive changes, then it might be due to development of skills other than visuospatial integration. Herein, we found microstructural adaptations in bilateral IFOF to be correlated with RPM score and subjects level of training in chess.

### Inferior longitudinal fasciculus

ILF fiber bundle links the occipital lobe with the anterior portion of the temporal lobe, working spatially and functionally close to IFOF (Ashtari, 2012). This bundle is known to be involved in object recognition tasks (Ortibus et al., 2012), and its disruption is implicated in visual memory loss (Shinoura et al., 2007). More recent studies suggest a specific object-naming role for left ILF (Shinoura et al., 2010). In line with an association of ILF microstructure with levels of chess training in our study, a recent study investigated the perceptual components of chess expertise, suggesting an essential capability in professional chess players to rapidly recognize complex visual patterns (Sheridan and Reingold, 2017).

### Cingulate gyrus

Cingulate gyrus is a complex and multifunctional brain region, associated with various cognitive abilities. Functional imaging studies have previously demonstrated a role for cingulate gyrus in complex decision-making, comprising both cognitive and emotional areas, and regulating emotional decisions (Posner et al., 2007).

The anterior cingulate cortex is highly active during tasks relating attitudes to decision making and tasks requiring multi-domain executive function (Shenhav et al., 2016). The ACC is a major node in the “face recognition network,” adding attitudes and mental state values to the face-processing signals (Haist et al., 2013). A recent study suggested a role for dorsal anterior cingulate cortex in maintaining internal goals and suppression of distracting behaviors (Shenhav et al., 2016).

Posterior cingulate gyrus is, in turn, an essential part of default mode network and is strongly involved in self-awareness and internal goal-directed behavior (Leech and Sharp, 2014). One study demonstrated increased activity in posterior cingulate gyrus parallel with extensive training and proficiency in visuomotor learning tasks in football players (Morgan et al., 2016). Bilateral anterior and posterior parts of the cingulate gyrus are active during memory-related tasks in chess players but not novices (Campitelli et al., 2005). Finally posterior cingulate gyrus is activated during activities associated with processing of game configurations i.e., distinguishing chess boards from random boards (Krawczyk et al., 2011). Interestingly this area was not active during recognition of other visual stimuli and was a chess-related cognitive area (Krawczyk et al., 2011). Finally, and in line with the known role of posterior cingulate gyrus in goal-directed behavior, posterior cingulate conducts a series of self-directed thinking about chess in professional players, but not novices, when chess boards were displayed (Krawczyk et al., 2011).

Herein we found higher planar anisotropy of bilateral cingulate gyri in professional chess players along with strong positive correlation of FA of cingulate gyri with subjects RPM score and a decrease in the MD values with an increased in the level of training in professional players. Results of the DMRI connectometry also revealed difference in connectivity of bilateral cingulum is professional players. Cingulum entails fibers in close proximity and entails afferents/efferents to and from the cingulate gyrus. Putting the above evidence with domain specific abilities required in professional chess practice (Burgoyne et al., 2016), a distinct role for cingulate gyrus in adaptive structural changes in chess players brain is demonstrated.

### Superior longitudinal fasciculus

SLF was found by Hanggi et. al. to undergo structural adaptations in professional players while having a direct correlation with players Elo's score and hours of practice (Hanggi et al., 2014). We demonstrated that diffusometric variables of bilateral SLF were correlated with the level of chess plying and professional training time.

Professional chess players also demonstrated a concurrent increase in connectivity in the left temporal part of SLF (SLF IV or arcuate fasciculus) (Table 1). Arcuate fasciculus is a language comprehension area with involvement in semantic skills (Broce et al., 2015). Association of these temporal connections of SLF with expertise in chess playing remains to be more elucidated.

### Corpus callosum

As the brain's largest WM tract, CC is speculated to play a major role in cognitive tasks. CC appears to contribute to a declined cognition in aging adults (Chepuri et al., 2002). Keeping with the role of CC in cognition and executive function, it has been revealed that individuals with agenesis of corpus callosum have poorer behavioral performance in specific cognitive tasks (Hinkley et al., 2012), associated with a general loss of connectivity in both inter- and intrahemispheric areas. These facts are in line with the results of our connectometry analysis finding increased connectivity in genu, body, and splenium of CC in professionals compared to novices.

### Structural underpinning of expertise in chess-players brain

Chess training is related to a variety of cognitive benefits, especially when started early and extensively at early school age (Sala et al., 2017). Cognitive benefits are moderate and mainly involve mathematical achievements and overall cognitive ability (Sala et al., 2017). These effects have been linked to “far transfer,” which explains how expertise in one area can improve basic skills that prove useful in other cognitive domains or unrelated areas (Sala and Gobet, 2016). It is thus plausible that underpinning microstructural adaptations in chess-players are observed in other expertise domains (Bengtsson et al., 2005), that are not necessarily active during chess playing. With sparse evidence and uncertainty in the design of such studies, understanding microstructural adaptations in a chess-players brain could help elucidate cognitive benefits of chess training in the developing brain. Neuroplasticity during active chess-related training is possibly the primary underlying mechanism for the observed differences in bilateral IFOF, bilateral ILF and cingulate gyrus between professional players and also the robust correlation of diffusion parameters in bilateral uncinate fasciculi and bilateral anterior thalamic radiations with training level and time.

As a final remark, although studies have clearly stated that increased FA and decreased RD are indicators of axonal coherence and fiber health, there are inconsistent results on change of diffusion indicators associated with neuroplasticity (Zatorre et al., 2012). This could further explain why not all diffusometric parameters showed correlation with training time and level of expertise in chess players and also explains partly, the inconsistencies in the results of our analyses in each step.

## Conclusion

This study is the first to investigate alterations in whole brain diffusivity and structural connectivity that happen with professional chess playing, using both DMRI region-of-interest and connectometry approaches. We demonstrated that these adaptations directly correlated with level of chess playing and time spent on training chess.

Region-of-interest analysis of DTI data revealed increased WM integrity in bilateral anterior thalamic radiations, bilateral cingulum, bilateral ILF, and bilateral UF and right hippocampus in professional players compared to novices.

Partial correlation analysis confirmed the above findings as, bilateral ILF showed increased fractional anisotropy and planar anisotropy in professional players, both correlated with higher score in RPM test. This was compatible with fiber maturation and structural integrity that happen with chess practice. Higher fractional anisotropy and planar anisotropy, concomitant with reduced axial diffusivity in left ILF, correlated with longer duration of professional training in chess players. A concurrent increase in fractional and planar anisotropy of right anterior thalamic radiations and right UF occurred with an increase in professional training duration in chess players. Diffusion MRI connectometry revealed increased structural connectivity in bilateral UF, bilateral IFOF, bilateral cingulum, right arcuate fasciculus and corpus callosum in professional players compared to controls. Together our findings reiterate that the observed changes in regional volume and function that happen with professional training in chess enroot from structural adaptations, in terms of diffusivity and microstructural connectivity, in several white and gray matter fibers. These adaptations are the direct result of training and mastership in playing chess.

## Author contributions

MM: conceptualized and drafted the manuscript and performed complementary analyses; MHA: conceptualized, analyzed the database and helped writing the methods, results and draw DMRI figures; FR: helped in interpretation of the results, drafting the manuscript and its critical revision.

### Conflict of interest statement

The authors declare that the research was conducted in the absence of any commercial or financial relationships that could be construed as a potential conflict of interest.
